# A Review of Metal Nanoparticles Embedded in Hydrogel Scaffolds for Wound Healing In Vivo

**DOI:** 10.3390/gels9070591

**Published:** 2023-07-22

**Authors:** Sara Sheikh-Oleslami, Brendan Tao, Jonathan D’Souza, Fahad Butt, Hareshan Suntharalingam, Lucas Rempel, Nafise Amiri

**Affiliations:** 1 Faculty of Medicine, The University of British Columbia, 317-2194 Health Sciences Mall, Vancouver, BC V6T 1Z3, Canada; 2 Faculty of Engineering, McMaster University, 1280 Main Street West, Hamilton, ON L8S 4L7, Canada; 3 Faculty of Science, McMaster University, 1280 Main Street West, Hamilton, ON L8S 4L7, Canada; 4 International Collaboration on Repair Discoveries, 818 West 10th Avenue, Vancouver, BC V5Z 1M9, Canada

**Keywords:** nanoparticles, nanotechnology, wound healing, antimicrobial agents, hydrogels

## Abstract

An evolving field, nanotechnology has made its mark in the fields of nanoscience, nanoparticles, nanomaterials, and nanomedicine. Specifically, metal nanoparticles have garnered attention for their diverse use and applicability to dressings for wound healing due to their antimicrobial properties. Given their convenient integration into wound dressings, there has been increasing focus dedicated to investigating the physical, mechanical, and biological characteristics of these nanoparticles as well as their incorporation into biocomposite materials, such as hydrogel scaffolds for use in lieu of antibiotics as well as to accelerate and ameliorate healing. Though rigorously tested and applied in both medical and non-medical applications, further investigations have not been carried out to bring metal nanoparticle–hydrogel composites into clinical practice. In this review, we provide an up-to-date, comprehensive review of advancements in the field, with emphasis on implications on wound healing in in vivo experiments.

## 1. Introduction

Wound healing is an intricate physiological process consisting of a series of molecular and cellular events that facilitate the regeneration of the skin, a protective barrier against the external environment. Since its inception, hydrogels have advanced the field of wound healing, insofar as to promote damaged tissue healing within a hydrated milieu [1]. As well, the integration of therapeutic nanoparticles (NP) and biomolecules into hydrogels for local wound application has been shown to enhance and accelerate healing [2,3]. In this era of increasing antibiotic resistance, nanoparticles with antimicrobial properties are mainstay alternatives to the incorporation of antibiotics into hydrogels. The literature also contains evidence for the application of other inclusions such as metals, growth factor-releasing nanoparticles, and enzyme-releasing nanoparticles. Continuing advancements in hydrogel synthesis and nanoparticle integration offer a vast range of possibilities for creating more effective therapeutic gels. 

Chronic wounds are increasingly a cause of public health concern. Indeed, under the growing population of patients with obesity and advanced age, the burden of pressure sores, venous insufficiency, and diabetes is expected to increase along with secondary instances of chronic wounds [4]. As such, a corresponding rise in treatment costs for chronic wounds is expected to strain current systems of healthcare delivery [5]. If undertreated, the persistence of chronic wounds can lead to delayed healing, progression of infectious wound infiltration, and reduced patient quality of life [5]. This outcome is especially likely in underserved populations, thereby grounding chronic wounds as a growing global health issue. Hydrogels are uniquely positioned to offer cost-effective and optimized local treatment for chronic wounds; moreover, they are non-toxic and non-irritant, biocompatible, easily applicable, and cost-effective [6]. Therefore, additional research and literature synthesis are required to support the calculated expansion of this rapidly evolving field.

Of importance, the incorporation of metal nanoparticles into hydrogels has been shown to enhance antimicrobial properties and accelerate healing [7]. Silver nanoparticles are a notable example that have demonstrated efficacy in reducing bacterial growth to promote wound healing [6,7]. One study reporting the application of guar gum hydrogels with embedded silver nanoparticles as wound dressings in a rat model demonstrates > 40% wound healing in the setting of 60% antibacterial efficacy, altogether with only 20% local cytotoxicity, after 12 days post-incision [8]. Another study reports on the spectrum of antimicrobial activity of silver nanoparticle inclusions in Swiss Albino mice, noting rapid healing with insignificant scarring after 48 h, exhibiting a stronger bactericidal selectivity against *S. aureus* than *E. coli* (92% overall bacterial reduction) [9]. Another report demonstrates additional properties of silver nanoparticles such as enhanced wound re-epithelialization, cell proliferation, and reduced tissue inflammation [2]. Zinc is another example of a metal nanoparticle that has exhibited antimicrobial properties when incorporated into hydrogel scaffolds. Similarly efficacious results with respect to antimicrobial activity and wound healing have been demonstrated with gold nanoparticle applications [10]. Although beyond the scope of this review, metal nanoparticles embedded in hydrogels have been studied for applications other than wound healing, such as for drug delivery, tissue engineering, cancer treatments, and imaging, each of which deserves further study [11]. Nonetheless, the application of metal nanoparticles for wound healing shows tremendous promise for clinical application to accelerate and ameliorate wound healing. However, further research is required to elucidate the therapeutic capabilities of these nanoparticles through the optimization of their design and delivery to wounded tissue. With recent advances in the application of metal nanoparticles into hydrogel scaffolds, there is a need to summarize new results to inform promising future areas of research. The rationale for this study was therefore to summarize the effects of metal nanoparticle-embedded hydrogel scaffolds with antimicrobial properties for wound healing in the literature on in vitro and in vivo results. 

The primary objective of this review was to identify the corpus of nanoparticle-embedded hydrogels that, to date, have been investigated for their antimicrobial properties in wound healing. The secondary objective was to synthesize current evidence on key therapeutic properties as they pertain to each nanoparticle agent. 

## 2. Methods

This systematic review abides by Preferred Reporting Items for Systematic Reviews and Meta-Analyses. The protocol for this review was prospectively registered with PROSPERO (identifier: 375393). As recommended by article 2.4 of the Tri-Council Policy Statement, IRB/Ethics Committee approval was not required for this study since data were extracted from published primary research. This research adheres to the tenets of the Declaration of Helsinki, and where appropriate, adheres to PRISMA reporting guidelines.

### 2.1. Eligibility Criteria for Considering Studies for This Review

We included any in vivo study that investigated the effect of metallic nanoparticles embedded in hydrogel scaffolds to enhance antimicrobial properties during wound healing. No preference was discerned for exposures, such as infection-induced pathogens. There were no restrictions on the type of hydrogel scaffolding. Studies were excluded if they investigated non-metal nanoparticle agents, such as polymeric or solid lipid nanoparticles. Additionally, studies focusing on other parameters of hydrogel efficacy, such as the promotion of angiogenesis, were not recommended for study inclusion. Non-English-written studies were also excluded.

### 2.2. Search Methods for Identifying Studies

**Figure 1:**
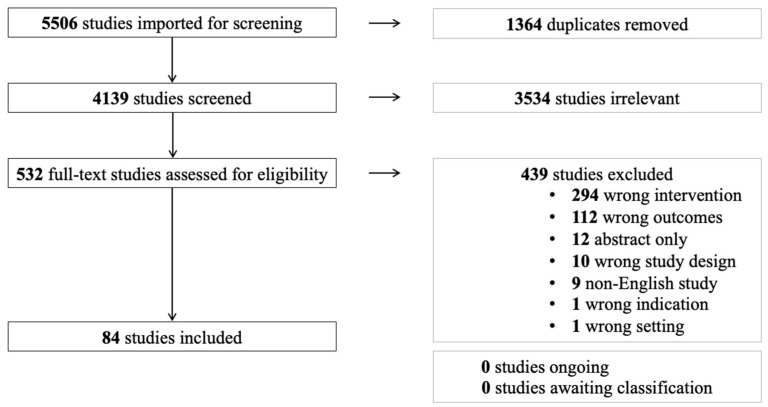
PRISMA flow diagram for study inclusion.

On 30 October 2022, Ovid MEDLINE and EMBASE were searched by SS using the study eligibility criteria. The search strategy was developed in consultation with an institutional librarian. Notable search restrictions included the English language. Study record management and eligibility assessment were completed in Covidence (Melbourne, Australia). Figure 1. depicts the PRISMA flow diagram for study inclusion for this review. 

Excluded studies fell under the following categories: wrong intervention, where studies did not include metal nanoparticles embedded in hydrogel scaffolds; wrong outcomes, where studies did not include review wound healing outcomes; wrong indication, where studies did not have the primary objective to investigate the effect of the intervention on wound healing; wrong study design, where in vivo investigations were not conducted; abstract only; non-English studies; and wrong setting, where studies discussed hydrogel preparation.

### 2.3. Data Collection and Risk of Bias Assessment

Title and abstract screening were completed by two independent reviewers (FB and JD) using Covidence (Melbourne, Australia). Studies were excluded if they were in vitro studies or if they did not describe wound healing outcomes with hydrogel scaffolding. Full-text articles were subsequently evaluated by two independent review authors (FB and JD), with reasons documented for study exclusion. Data extraction proceeded independently and in duplicate (FB, HS, JD, and LR). Variables recommended for extraction included hydrogel types; notable structural and in vitro properties; antimicrobial agent delivered; particle size; dosage delivered; rate of release; antimicrobial capacity; inhibition zone; cell type; cytotoxic effects; validated animal model; application method for wound healing; and year of publication. Conflicts at any stage in these review steps were adjudicated by a lead study author (SS and BT). For title and abstract screening, full-text evaluation, and data extraction, all review authors (SS, BT, FB, and JD) evaluated the first 100 records to calibrate inter-rater inconsistencies. Risk of bias evaluation was not conducted as the included studies were overwhelmingly conducted in a controlled non-clinical setting. 

### 2.4. Data Synthesis and Analysis

The primary objective of this review was to produce a summary catalogue of nanoparticle-embedded hydrogels that, to date, have been investigated for their antimicrobial properties in wound healing in vivo. The secondary objective was to synthesize the evidence of study findings for each nanoparticle agent. A thematic analysis approach was used to summarize nanoparticles features as they relate to hydrogel types; notable structural and in vitro properties; antimicrobial agent delivered; particle size; dosage delivered; rate of release; antimicrobial capacity; inhibition zone; cell type; cytotoxic effects; validated animal model; application method for wound healing; and year of publication. The thematic analysis was reviewed by all study authors. All results that were applicable to each secondary outcome were sought from each study. 

## 3. Wound Healing

### 3.1. Normal Wound Healing

In order to understand aberrant healing, normal wound repair and healing processes must be understood. Defined as an injury to the body involving laceration or breaking of a membrane usually with damage to underlying tissues, a wound can be acute or chronic [12]. In the acute phase of normal wound healing, there are four principal phases: hemostasis, inflammation, proliferation, and remodeling. Various cell types are involved in the healing process, such as fibroblasts and keratinocytes as well as proteins, hormones, cytokines, and enzymes [13]. 

Immediately following injury, there is bleeding. In the hemostatic phase of wound healing, vasoconstriction of arterial vessels occurs. Subsequent hypoxia and acidosis within the wound result in relaxation of the blood vessels with bleeding ensuing once more. Exposure of blood to the subendothelial tissues allows for the activation of the proteolytic cleavage and clotting cascades [14]. Platelets aggregate to form a hemostatic plug, releasing growth factors such as platelet-derived growth factor (PDGF), transforming growth factor-ß (TGF-ß), epidermal growth factor (EGF), and insulin-like growth factors (IGF), which trigger angiogenesis and activate and fibroblasts, endothelial cells, macrophages, and neutrophils [15]. Serotonin, histamine, and other vasoactive amines are released by mast cells and platelets, resulting in vasodilation and increased vascular permeability [15]. 

The inflammatory phase begins shortly after, with the activation of the complement cascade resulting in the attraction of leukocytes to the site of injury. Neutrophils and macrophages play a role in the removal of bacteria, damaged tissue, and foreign bodies from the site, thereby preventing infection [16]. Activation of macrophages results in further release of growth factors, which initiate subsequent cellular reactions [17]. Lymphocytes are attracted at 72 h post-injury [15].

The proliferation phase begins 72 h to approximately 2 weeks post-injury. Fibroblasts migrate and deposit extracellular tissue matrix (ECM), including fibrous proteins, collagen, polysaccharides, proteoglycans, glycosaminoglycans, and fibronectin, which appear as granulation tissue [18]. During this time, collagen synthesis and deposition simultaneous occur, with the collagen type dependent on the nature of the injury and healing factors present [15]. Wound contraction occurs by myofibroblasts via interactions with the ECM through the shrinking of connective tissue, effectively bringing wound edges closer together [19]. 

Lastly, the remodeling phase occurs, which may extend beyond 1–2 years [15]. Collagen bundles increase in diameter and become more organized within the matrix and matrix metalloproteinase enzymes are reduced [15,19]. 

### 3.2. Delayed Wound Healing 

In healthy individuals, standard healing of acute wounds takes approximately 2–3 weeks followed by the remodeling phase, which takes 1–2 years [16]. Chronic wounds are generally defined as those taking longer than 6 weeks to heal or that have frequent recurrence [4]. As wound healing is an intricate process, a deviation in the physiological healing process may result in arrest in one of the four phases, resulting in a halt of healing progression. Many factors can contribute to delayed healing, including but not limited to chronic disease, wound infection, persistence of foreign bodies, vascular insufficiency, diabetes, neurological deficits, nutritional deficiencies, chronic irritation and trauma [4]. With chronic wounds, pathological damage extends beyond the area of compromised skin integrity, resulting in pathological damage to surrounding tissues as well [20].

### 3.3. Infected Wound Healing 

As the majority of wound beds are moist, warm, and nutritious, they are highly susceptible to infection and provide optimized environments for the growth and colonization of bacteria [21]. The most predominant species found in infected wounds include *Staphylococcus aureus*, *Pseudomonas aeruginosa*, *Escherichia coli*, *Enterobacter cloacae*, *Klebsiella* species, *Streptococcus* species, and *Proteus* species [22]. Bacteria are present on virtually all open wounds [4]. When the growth of bacteria does not exceed host defenses, wound beds are considered colonized and may actually hasten wound healing via the promotion of wound bed perfusion [23]. Once host defenses are no longer able to maintain the balance between bacterial growth and death, the wound becomes infected and enters a non-healing state [23]. Infected wounds are often polymicrobial, with *Staphylococcus aureus* and anaerobes being the most common [22]. Infected wounds pose a burden to both the patient as well as the health care system due to increased costs of care [24]. 

Most commercial products are not catered towards wounds with active infection. Such wounds progress to requiring systemic antibiotic therapy; however, this also has complications such as systemic toxicity, associated with end organ damage [25]. Additionally, systemic therapy does not penetrate well into ischemic or necrotic tissues. The use of local antibiotics, usually topically, has grown more popular, with Acticoat, high-density polyethylene mesh with nanocrystalline silver coating, as a prime example [21]. However, with the increasing use of antibiotics, antibiotic-resistant microorganisms have considerably increased [26]. This is an issue encountered both systemically and topically. As such, new strategies have been sought out to curb wound infections without the risks of antibiotics as listed above. 

## 4. Current Wound Dressing Materials and Limitations 

There is a longstanding history of wound dressings that dates back to ancient times, all of which demonstrate designs to manipulate the wound environment for the facilitation of wound healing [4]. Today, a myriad of wound care products are available, all majorly created with the understanding that a moist environment is crucial for cell migration and the facilitation of wound healing and contraction [16]. Further, these products aim to remove excess exudate, minimize trauma on removal, and protect against contaminants [4]. Currently, the following dressing materials described below are available [4]: 

**Peri-wound protection**: Protects the integrity of the tissue surrounding the wound; includes ointments and barrier creams. 

**Gauze**: Provides absorption but promotes desiccation of the wound base, which hinders healing. Moreover, binding of the gauze to the wound bed often results in trauma upon removal [27]. Moreover, as gauze dressings can become fully saturated with wound exudate, they are not effective barriers against infection. Modern gauze dressings have substances embedded to optimize the wound environment. 

**Films**: Adhesive, non-absorbent, semi-occlusive dressings that can be used as primary or secondary dressings that facilitate the exchange between oxygen and water vapor between the wound bed and the external environment while still providing barrier functions against infection.

**Foams**: Non-adhesive, absorbent, semi-occlusive dressings that protect against shear with non-traumatic application and removal. 

**Hydrogels:** Water-based, facilitating moist wound-healing environments. Application and removal cause minimal trauma to the wound bed. Further, hydrogels promote autolytic debridement. 

**Hydrocolloids**: Adhesive, absorbent, occlusive dressings that are gel-forming, able to absorb large amounts of exudate while still maintaining moist wound-healing environments.

While wound dressings shield wounds from external contamination, functionalization and delivery of antibiotic properties directly to the wound bed have been investigated in efforts to accelerate and ameliorate the healing of infected wounds [28]. The incorporation of bioactive agents with such dressings has grown in popularity with increasing potential to be used for wound treatment. Due to their localized effect, this method is postulated to be more effective in the medicinal treatment of non-healing chronic wounds [29]. Despite this, the direct application of bioactive agents is still found to be more effective due to faster absorption [30].

## 5. Biopolymers in Wound Healing

As previously described, many different varieties of antibacterial wound dressings have been developed for wound treatment, including gauze, films, foams, hydrocolloids, alginates, collagens, and hydrogels. One issue with common wound dressings is their propensity to adhere to the wound site and edges, disrupting healing processes and causing damage to the wound bed [31]. Hydrogels, three-dimensional scaffold networks, are promising dressings as they provide suitable environments for cellular growth and adhesion [6]. They have high water content and also swell in hydrated environments, thus being able to absorb wound exudate while still maintaining a moist wound environment [4]. As hydrogels can retain large amounts of water, they are flexible and elastic [31]. Another issue with common scaffolds is that they have to be implanted at the wound bed, whereas hydrogels are able to conform to wound topography, filling all defects of the wound from the bottom up [6]. 

Hydrogel dressings can be made from many materials. Most commonly, polymers of synthetic molecules are used, such as polyacrylamide or polyvinylpyrrolidine [6]. These polymers are non-toxic and can maintain their shape; however, they have limited tissue adhesion properties and, as such, must be combined with other materials for the adjustment of their mechanical properties [31]. 

Another commonly used biopolymer is cellulose, a component of plant cell walls that is cost-efficient and ameliorates wound healing through the release of growth factors to stimulate granulation tissue formation, re-epithelialization, and angiogenesis as well as proliferation and movement of fibroblasts to the wound bed [32,33]. While more costly, chitin is another frequently used polysaccharide biopolymer for the formation of hydrogels [34]. Chitin is transformed into chitosan, which is soluble in aqueous solutions. In addition to its strong mechanical properties for wound healing, it also offers some antimicrobial properties as it is a cation, thus being able to treat infections through the disruption of cell membranes [35,36]. 

Alginate is another biopolymer that can be used in the preparation of hydrogels, but also in other dressings such as foams due to its absorbent properties [37,38,39,40]. It has good cytocompatibility and is non-toxic. It can be used in combination with other biopolymers, such as synthetic polymers, to enhance the biological and mechanical properties of the 3D hydrogel scaffold [41,42]. Gelatin, another naturally occurring polymer, is derived from collagen and is also used for its biocompatibility and biodegradable properties [43]. Likewise, fibrin, derived from fibrinogen, offers similar properties when used for wound healing, including reduction in inflammation, promotion of cell adhesion properties, and immunomodulation [43].

Starch, another biopolymer derived from plants, comes from various sources in many different shapes and sizes [44,45,46,47]. Apart from application in hydrogel scaffolds, given their cytocompatibility, low toxicity, and biodegradable nature, starches can be used in many other applications, including in drug delivery systems [44,45,46,47]. 

All the polymers mentioned above, both biological and synthetic, have been used in conjunction with metal nanoparticles for an enhanced healing effect. Other notable materials observed to be used in such combinations include dextran, elastin, silica, tannic acid, and lignin.

## 6. Nanotechnology for Wound Healing 

Nanotechnology is defined as the manipulation of materials on an atomic or molecular scale [48]. Ever evolving, nanotechnology has revolutionized many industries, especially within the fields of nanoscience, nanoparticles, nanomaterials, and nanomedicine. Specifically, the field of nanomedicine has risen in popularity with myriad applications, including vaccine production, wearable devices, implants, drug delivery, and antibacterial applications [49]. In tissue engineering and regenerative medicine, nanomaterials have shown low toxicity and customizability, making them versatile agents to incorporate into medical practice [49]. For instance, metal nanoparticles such as silver (Ag) [50], gold (Au) [51], copper (Cu) [52], and zinc oxide (ZnO) [53] have demonstrated marked antimicrobial properties. While these intrinsic properties are advantageous for wound healing, these metal nanoparticles can also display anti-infective properties within drug-delivery vehicles. 

## 7. Nanoparticles Used in Wound Healing

Metal nanoparticles have been considered in clinical applications for reasons including small size, high surface-to-volume ratio, shape, stability, low toxicity, and economic reasons, given their affordability [54,55]. Additionally, they can conveniently integrate into wound dressings [54]. One of the primary mechanisms in which antibacterial activity is offered by metal nanoparticles is through their bacteriostatic properties via attachment to DNA or RNA, via electrostatic interactions, halting further replication [56]. MicroRNAs, short, non-coding RNA molecules that have regulatory roles in gene expression, play a large role in wound healing processes, including inflammation, angiogenesis, cell proliferation, and ECM remodeling. In aberrant wound healing, such as infectious states, microRNAs can be targeted by metal nanoparticles through encapsulation, shielding charge groups and allowing for cellular uptake [57]. The modulation of microRNA allows for the enhancement of gene expression factors, promoting the production of factors essential for wound healing. Further, targeted delivery of these therapeutic agents minimizes off-target effects [57].

**Figure 2:**
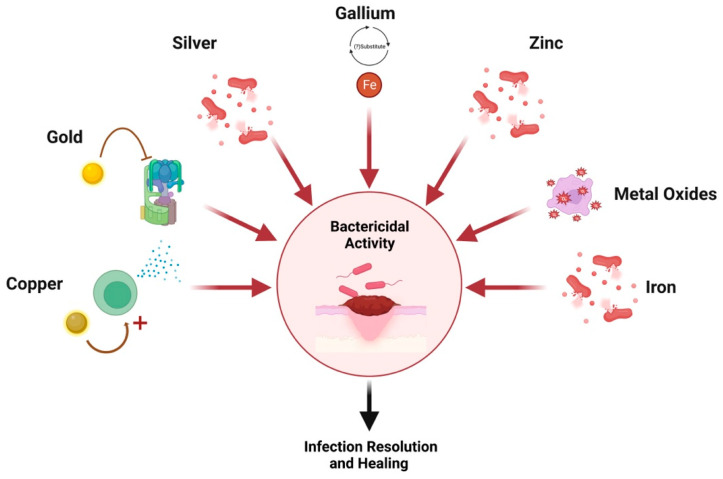
Nanoparticle mechanisms of action. Created with Biorender.com.

Another mechanism is through bactericidal properties via the creation of reactive oxygen species [56]. When embedded in hydrogel scaffolds, a substitute is created for damaged ECM which facilitates fibroblast proliferation and matrix formation for enhanced regeneration and repair [58,59]. As such, these nanoparticles can be used in lieu of antibiotics and are thought to accelerate and ameliorate healing while preventing infection [54,55]. A graphical summary of wound healing mechanisms per nanoparticle is depicted in Figure 2.

### 7.1. Silver (Ag) Nanoparticles

The use of silver for the treatment of wounds and infection prevention dates back to at least 4000 B.C.E. with documented medical applications dating back to the 1700s; however, in large quantities, silver can also impair healing due to its toxic effects on keratinocytes and fibroblasts [60,61]. Today, silver continues to serve many applications in wound healing. For example, silver nitrate is used as a commonplace treatment for chronic wounds while silver sulfadiazine is used for burns. Nanotechnology has changed the use of silver for wound healing with the creation of silver nanoparticles (AgNPs), which are the most commonly used metal nanoparticles in wound management with many applications, including wound infections, ulcers, and burns. Known for their wide range of antimicrobial activity, effective against bacteria, viruses, fungi, and protozoa, as well as promotion of wound healing, AgNPs have been shown to disturb quorum sensing, effectively reducing biofilm formation [62,63,64].

The antibacterial effect is demonstrated via bactericidal and inhibitory mechanisms. In terms of bactericidal activity, apoptosis is induced in bacteria through AgNP interactions with sulfur and phosphorous-containing proteins, effectively disrupting cell membranes [65]. Moreover, as DNA consists of sulfur and phosphorous, AgNPs act on these bases to destroy DNA, further facilitating the apoptosis of bacterial cells [65]. Moreover, the continuous release of AgNPs, specifically at lower pH whereby acidic environments facilitate the oxidation of AgNPs to Ag^+^, negatively charged proteins are bound to, allowing for disruption of bacterial cell walls and membrane [65]. Through this mechanism, cell respiration is also disrupted through damage to bacterial mitochondria [66,67]. Despite these cytotoxic effects, which are AgNP-dose- and size-dependent, the proliferation of fibroblasts and keratinocytes is not affected [68]. In terms of inhibitory mechanisms, the presence of AgNPs in the wound environment allows for the formation of reactive oxygen species (ROS), which further disrupt bacterial cell viability through oxidative stress [66,67]. Wound healing is accelerated through these antibacterial properties as microbes can delay all stages of wound healing. 

In addition to the antimicrobial properties of AgNPs, they also promote wound healing [69,70,71,72]. Firstly, they assist in the differentiation of fibroblasts into myofibroblasts, which allows for wound contractility [73]. Moreover, they stimulate the proliferation and relocation of keratinocytes to the wound bed [74]. As such, quicker wound epithelialization and scarless wound healing are promoted [73]. Accelerated and complete healing with increased epithelialization was observed in a study wherein an AgNP hydrogel was applied to a partial-thickness cutaneous wound in mice [75,76].

AgNPs also have anti-inflammatory effects through cytokine modulation, reducing levels that allow for decreased lymphocyte infiltration, further enhancing re-epithelialization [52,77]. One study demonstrated a significant reduction in inflammatory cytokines and oxidative stress, effectively promoting healing, while another study in a burn wound model in mice demonstrated reduced interleukin-6 (IL-6) and neutrophils and increased the levels of IL-10, vascular endothelial growth factor, and TGF-ß [50]. 

The summary of all in vivo studies related to AgNP-loaded hydrogels is shown in Table 1.

### 7.2. Gold (Au) Nanoparticles

AuNPs are commonly used in tissue regeneration, wound healing, and drug delivery of bioactive compounds due to their biocompatibility, high surface reactivity, and antioxidative effects [118,119]. While some antimicrobial effects are seen, unlike AgNPs, AuNPs do not offer much antimicrobial activity alone [120]. 

Antimicrobial action is demonstrated via two principal mechanisms, similar to AgNPs: bactericidal and inhibitory. Cell death is induced via the disruption of ATP synthase, leading to decreased ATP stores and an eventual collapse in energy metabolism [121]. This is due to the ability of AuNPs to alter membrane potential on entry into the cell [121]. Additionally, the creation of ROS is facilitated by AuNPs, further facilitating cell death. The smaller the size of the AuNPs, the greater the surface area and interface for interaction with microbes, demonstrating a stronger antimicrobial effect [122].

While some antibacterial effects are seen, AuNPs are principally used in tissue repair given their anti-inflammatory properties via cytokine modulation and antioxidant properties [123,124]. Substantial antioxidant properties are seen as AuNPs are able to bind free radicals such as nitric oxide (NO) or hydroxyl (OH-) [125,126,127]. This strong catalytic activity in free radical scavenging is further observed through the ability of AuNPs to increase nuclear factor erythroid 2-related factor (NRF2), which allows for antioxidant gene activation [128,129]. Furthermore, while being able to facilitate the creation of ROS, they are also able to receive electrons and remove or deactivate ROS, with greater effects seen the higher the surface area of the AuNPs is [119]. 

In addition to tissue repair, wound healing is found to be accelerated and ameliorated with the use of AuNPs through the promotion of collagen expression, growth factors, vascular endothelial growth factor (VEGF), fibroblast proliferation, decreased cellular apoptosis, and angiogenesis [67,130]. 

Despite these beneficial effects, AuNPs must usually be incorporated with other biomolecules for efficacy in wound healing applications. Examples include the incorporation of AuNPs in chitosan or gelatin for the enhancement of wound healing or in collagen for a similar effect [51,52]. One study of a rat full-thickness excisional wound model demonstrated accelerated healing and wound closure with improved hemostasis and re-epithelization compared to the Tegaderm dressing and pure chitosan hydrogel controls in a chitosan-AuNP hydrogel [131]. Recent studies have also incorporated phototherapy in conjunction with AuNPs to achieve antimicrobial activity [53,132]. 

The summary of all in vivo studies related to AuNP-loaded hydrogels is shown in Table 2.

### 7.3. Copper (Cu) and Copper Oxide (CuO)

Previous studies have demonstrated that CuNPs have antimicrobial activity as well as properties that facilitate tissue repair. CuNPs have shown antibacterial activity against bacterial strains such as *Escherichia coli* and *Staphylococcus aureus* but also fungicidal effects [138,139,140,141,142,143]. The principal mechanism of action is through adhesion of the CuNP to bacteria due to their opposing electrical charges, resulting in a reduction reaction that weakens and destroys the bacterial cell wall. CuNPs have also shown antibacterial activity through the enhancement of immunity with the promotion of interleukin-2 (IL2) production, as well as its ability to serve as a cofactor for various enzymes such as cytochrome oxidase [144]. Additionally, CuNPs have an influence on cytokine regulation, thus also having anti-inflammatory properties [145]. In terms of tissue repair, ECM synthesis is promoted through the stimulation of ECM components such as fibrinogen and fibroblasts as well as the production of integrins and collagen [146,147]. One study observing the use of a CuNP-embedded hydrogel in the treatment of full-thickness excisional wounds in rats demonstrated an accelerated wound healing rate [52]. Despite these positive effects, CuNPs are prone to rapid oxidation, promotion of the production of free radicals, and instability, thus limiting its use [148,149]. 

CuO NPs have been used in multiple biomedical settings, such as in drug delivery, as anti-cancer agents, and wound healing given their biocompatibility, low toxicity, and antimicrobial properties [150,151]. The specific mechanism for the antibacterial effects of CuO remains unknown; however, it is postulated that it is related to the generation of ROS within bacterial cells [152]. However, with CuO NPs, antibacterial activity was partially related to bacterial properties. For example, different effects were noted with increased bactericidal activity in Gram-negative organisms, such as *E. coli*, compared to Gram-positive organisms, such as *S. aureus* [153]. Despite these antibacterial properties, one concern is toxicity, the induction of oxidative stress, and subsequent DNA and mitochondrial damage [154,155]. 

The summary of all in vivo studies related to Cu and CuO NP-loaded hydrogels is shown in Table 3 and Table 4, respectively.

### 7.4. Zinc (Zn) and Zinc Oxide (ZnO)

Zn and ZnO NPs are some of the most commonly used NPs in wound healing applications due to their anti-inflammatory and antimicrobial properties [166]. As inorganic agents, they are more stable than their organic agent counterparts. They are also advantageous in their ability to remain within the wound bed for longer periods of time [166,167]. The antimicrobial effects of Zn and ZnO NPs are due to disruption of cell membranes and oxidant injury [166,167]. Zinc also serves as a cofactor for metalloproteinases and other enzymatic complexes, promoting migration of keratinocytes and regeneration of the ECM [166,167]. A previous study examining full-thickness wounds in a rat model showed accelerated and ameliorated healing compared to control with improved re-epithelialization as well as increased collagen deposition and tissue granulation [167]. Moreover, both Zn and ZnO NPs have demonstrated good biocompatibility and low cytotoxicity [166]. 

Like other NPs, the Zn and ZnO NP effect is dependent on the size, surface-area-to-volume ratio, and concentration of the NPs [168]. Smaller NPs have been shown to be more cytotoxic given their larger surface-area-to-volume ratio, whereas larger NPs demonstrate increased cytocompatibility [169]. In fact, a previous study demonstrated that ZnO NPs are highly compatible with fibroblast cells and promote their growth, migration, and adhesion [142]. 

Summaries of all in vivo studies related to Zn and ZnO NP loaded hydrogels are shown in Table 5 and Table 6, respectively.

### 7.5. Other Metal Oxides 

Metal oxide NPs include zinc oxide (ZnO), copper oxide (CuO), cerium oxide (CeO_2_), manganese oxide (MnO_2_), and titanium oxide (TiO_2_). These NPs have antioxidant properties and have been shown to facilitate wound healing through the restriction of ROS, inhibiting apoptosis [166,167]. 

#### 7.5.1. Titanium Oxide (TiO_2_)

A study of the antimicrobial effects of TiO_2_ NPs demonstrated little effect but showed accelerated wound healing in a full-thickness excisional wound model [183]. 

#### 7.5.2. Cerium Oxide (CeO_2_)

CeO_2_ NPs have the highest antioxidant activity of all NPs and are most active in the scavenging of free radicals [184]. This is due to the oxygen vacancies of CeO_2_, leading to the reduction of Cerium from Ce^+4^ to Ce^+3^, for example [184]. 

#### 7.5.3. Manganese Oxide (MnO_2_)

MnO_2_ is also a potential candidate to be used in nanoparticle–hydrogel composites. Known to relieve oxidative stress, MnO_2_ is able to catalytically decompose H_2_O_2_ into O_2_, thus effectively providing a targeted approach to hypoxic relief [185]. One study evaluating MnO_2_ nanoparticles in the healing of chronic diabetic wounds in vivo demonstrated the eradication of biofilms, attenuation of hyperglycemia, hemostasis, and the creation of an optimized wound environment which reduced inflammation, accelerated granulation tissue formation and re-epithelialization, and accelerated wound healing [185]. The summary of all in vivo studies related to MnO_2_ NP-loaded hydrogels is shown in Table 7.

### 7.6. Iron (Fe) Nanoparticles (FeNP)

Less commonly used in antibacterial wound dressing applications, iron nanoparticles have been shown to induce bacterial death, membrane damage, DNA degradation, and lipid peroxidation [187,188,189]. One study demonstrated high antibacterial activities against *S. aureus* and *E. coli* both in vitro and in an in vivo infected full-thickness excisional wound model in mice where accelerated wound healing and anti-inflammatory properties were observed [187]. 

The summary of all in vivo studies related to FeNP-loaded hydrogels is shown in Table 8.

### 7.7. Gallium (Ga) Nanoparticles (GaNP)

Gallium is very infrequently used in wound healing applications. Given that gallium and iron have equal ionic radii, one study hypothesized that the substitution of iron with gallium would impair bacterial iron metabolism and exert an antimicrobial effect [194]. This has previously been observed in vitro, whereby gallium resulted in reduced bacterial survival [194,195,196]. Moreover, given the inability of gallium to be reduced in physiological environments, a property not shared with iron, gallium also disrupts enzyme activity [197]. A 2022 study by Qin et al. demonstrated the good antimicrobial effect of gallium embedded in an alginate-base hydrogel, with good biocompatibility against NIH3T3 cells in vitro as well as accelerated wound healing with good biocompatibility, angiogenesis, and collagen deposition compared to the control in an *S. aureus* infected full-thickness excisional wound model in mice [194]. 

The summary of all in vivo studies related to GaNP-loaded hydrogels is shown in Table 9.

### 7.8. Combinations of Metal Nanoparticles

Occasionally, metal NPs can be used in conjunction with each other to provide synergistic antibacterial effects. For example, one study investigated the effects of AgNP and CuNP within a chitosan hydrogel, demonstrating good antibacterial activity against *S. aureus* and *E. coli* with good biocompatibility and accelerated healing compared to control in an *S. aureus* infected full-thickness excisional wound model in type 1 diabetic rats [198]. Another study looked at the synergy between ZnO and AgNPs, demonstrating an excellent bactericidal effect against *E. coli* and *S. aureus* [199]. Interestingly, AgNPs were observed to exhibit a small amount of cytotoxicity when tested against mouse calvarial (MC3T3-E1) cells alone, but when used in conjunction with ZnO NPs, lower cytotoxicity was seen [199]. In an *S. aureus* infected partial thickness wound model in rats, the release of Ag^+^ and Zn^2+^ was found to stimulate immune function to produce a large number of white blood cells and neutrophils (2–4 times more than the control), thereby producing the synergistic antibacterial effects and accelerated wound healing [200].

The summary of all in vivo studies related to combinations of metal-NP-loaded hydrogels is shown in Table 10.

## 8. Challenges and Future Directions

While there are myriad advantages in the addition of metal NPs to hydrogel scaffolds, there are limitations to their use as well. Principally, cytotoxicity is of concern as the interactions of NPs and cells have not yet fully been elicited [65]. While exploration into the cytotoxicity of NPs has been investigated, the field lacks uniformity such that tests of different cell types have demonstrated different cytotoxic responses. Further, there are multiple factors that influence NP activity, including the size, shape, concentration within the hydrogel, and surface charge [93]. Another concern regarding NPs is release and uptake. Poor release or uptake limits the efficacy and quality of treatment provided whereas excess release can result in deposition in surrounding tissues or organs, toxicity, or other undesired effects [31,65,202]. For example, overuse of silver-containing dressings impair healing processes due to cytotoxicity against keratinocytes and fibroblasts as well as systemic adverse effects such as argyria [202,203,204]. Thus, despite an extensive repertoire of current studies, future investigations and development would benefit from further toxicity and cytocompatibility assessments both in vivo and in vitro and iterate on current formulations for hydrogels with metal nanoparticles. We also recommend continued translational research on the application of these dressings in clinical practice.

## 9. Conclusions

The main aim of this review is to highlight the antimicrobial activity of metal nanoparticles embedded within hydrogel scaffolds for wound healing. Here, we elaborately discussed how these nanoparticles interact with pathogens but also with the wound environment to eradicate infection and enhance the healing process. When used in conjunction, hydrogels and metal nanoparticles demonstrate a synergistic effect. Excellent activity was demonstrated by select metal nanoparticles embedded within hydrogels. Accelerated and ameliorated wound healing was also observed with the promotion of re-epithelialization, angiogenesis, increased deposition of collagen and granulation tissue, and downregulation of inflammatory processes. Despite the large body of research in the field, there is a gap in preclinical and clinical studies, which, if addressed, could facilitate the use of these dressings in common clinical practice.

## Figures and Tables

**Table 1:** Summary of Characteristics and Findings of Included Trials for Silver Nanoparticles.

Hydrogel Species	Particle Size (Diameter)	Dosage of NP	Rate of Release	Antimicrobial Capability	Cell Type	Cytotoxic Effect	Animal Model	In Vivo Findings	Year	Source
Polyvinyl Alcohol (PVA)	74.58 nm	10% *w*/*v*	-	Minimum inhibitory concentration 3.13 ug/mL for *E. coli* and 25 ug/mL for *S. aureus*	-	-	Full-thickness wounds in rabbits	Wound closure is accelerated with AgNP (12–14 days) compared to control with marketed drug (23 days)	2019	[78]
PVA and Cellulose	10–200 nm	1000 ppm	~42% after 48 h	More effectively inhibits *S. aureus* compared to *E. coli* (92% bacterial reduction)	-	-	Full-thickness wound in mice	Faster healing with minimum scarring was seen in the AgNP group	2016	[9]
Chitosan	10–20 nm	0.1 mg/g	Constant at regular time intervals	Inhibition zone: *Pseudomonas aeruginosa*: 21.47 ± 0.50 mm; *Bacillus cereus*:11.52 ± 0.19 mm *Staphylococcus aureus*: 19.97 ± 0.73 mm; *Escherichia coli*: 17.30 ± 0.03 mm; *Klebsiella pneumonia*: 15.50 ± 0.51 mm	Human leukemia cell lines (THP1)	Nontoxic with an IC50 of 151.10 μg/mL at 24 h	*P. aeruginosa* infected excisional wound model in rats	Faster healing with better scar appearance in AgNP group with lower bacterial counts and enhanced connective tissue production compared to control	2021	[79]
Guar gum	8.24 ± 4.20 nm	<0.200 nM	-	Day 12 post-incision colony count (cfu): AgNPs: 20 cfu; commercial antibacterial gel (control): 51 cfu	Human dermal fibroblast cells	Low cytotoxicity with 80% cells viability	Full-thickness wounds in rats	>40% wound healing and 60% antibacterial activity compared to commercial antibacterial gels	2021	[8]
Poly (ethylene glycol) diacrylate (PEGDA)	-	5, 25, and 30 mg/mL	15.2% Ag^+^ released in 4 h	Bacterial viability of *E. coli* and *S. aureus* after 5–10 min of NIR laser exposure is 0 Inhibition zone: 0 mm	L929 fibroblast cells	>90% cell viability for all groups other than the groups with 50 mg/mL	*S. aureus infected* excisional wound model in rats	Good sustained anti-bacterial effects observed with greater wound healing response in experimental group 7 days after treatment	2022	[80]
Polyacrylic acid	-	0.1% *w*/*v*		Effective against *S. aureus* and *E. coli*	Murine fibroblast 3T3 cells	Proliferation promoted; low toxicity and good cell viability	Full-thickness wound model in diabetic mice	97% wound reduction compared to 81% wound reduction for control on day 14	2022	[81]
Chitosan and gelatin	20–80 nm; mean 36 nm	1% *w*/*v*	-	Highest biofilm eradication noted against *S. aureus* and *P. aeruginosa* with AgNP (89 ± 5%)	Mouse embryonic fibroblasts	Low toxicity	Excisional wound splinting model in BALB/c mice	Accelerated and ameliorated wound healing compared to control with enhanced angiogenesis, collagen synthesis, and sebaceous gland/hair follicle regeneration	2022	[82]
PF127 polymer	33 nm	0.1, 0.3, and 1.0 mg	-	Zone of Inhibition: *Bacillus cereus* (17.7 mm), *Escherichia coli* (18.7 mm), *Pseudomonas aeruginosa* (10.3 mm), and *Staphylococcus aureus* (17.7 mm) Minimum inhibitory concentration and minimum bactericidal concentration: 390–780 µg/mL	Drosophila melanogaster eggs	No significant effect on eclosion of F1 flies for doses under 250 micrograms/mL of AI-AgNPs	Full-thickness excisional wound model in mice	In a concentration-dependent manner, the wound contraction for the treatment groups were higher than for the control group; no skin irritation observed in patch test	2021	[83]
Chitosan	20–35 nm	-	Continuous with release co-efficient of 0.37	Zone of Inhibition: *E. coli*: 13.6 ± 0.3 mm; *B. subtilis*: 10.5 ± 0.8 mm; *P. aeruginosa*: 9.2 ± 0.3 mm; *S. aureus*: 11.4 ± 0.1 mm	-	-	Full-thickness excisional wound model in diabetic rats	The rate of wound contraction was higher compared to the control. Scar-free regeneration of the skin with intact patches of hair growth was also noted with the AgNP treatment	2021	[84]
Polyvinylpyrrolidone (PVP), polyethylene glycol (PEG), carboxymethyl cellulose (CMC)	31 nm	-	-	-	NCTC L929 cell line	Non-cytotoxic	Full-thickness excisional wounds in rabbits	Stimulatory action on wound healing as evidenced by a high intensity of fibroblasts and neovascularization in the tissue, which promoted a faster healing process when compared to the untreated wounds	2018	[85]
Gelatin	68.5 nm	200 µg/mL	With irradiation: 39.24%; without: 29.92% after 30 min	22.46% of methicillin-resistant *Staphylococcus aureus* (MRSA) and 24.48% of *E. coli* were eliminated compared to 22.46% and 20.37% in the control without irradiation. With irradiation, 97.57% of MRSA and 95.99% *E. coli* were killed due to photothermal effects of AgNPs	HaCAT cells	At <250 µg/mL, cell viability >80%, however cell viability decreased with increased concentration	MRSA-infected wound model in mice	91.76% of MRSA in wounds was removed with improved healing, angiogenesis, and collagen deposition.	2021	[86]
Lignin and cellulose	100 nm	3, 6, 9, 12% *w*/*v*	-	When in contact with the 12% *w*/*v* AgNP hydrogel for 2 h, *E. coli*, *S. aureus*, *C. albicans*, and *MRSA* had no colonies on the agar plates, indicating that all bacteria that were in contact with the hydrogels were killed	Mouse L929 fibroblast cells	Survival rate of cells in each concentration group was >90%	Full-thickness excisional wound infected with MRSA in rats	The hydrogels can maintain a moist healing environment, reduce inflammatory cell infiltration, promote M2 macrophage polarization, accelerate collagen deposition, promote angiogenesis, and accelerate wound healing of MRSA-infected wounds.	2021	[87]
Alginate and Gelatin	7.5–8.3 nm at 1 mM, 20–34 nm at 4 mM	1.0, 2.0, 40.0 nM	-	**Minimum Inhibitory Concentration**: 0.50 µg/mL against *Pseudomonas aeruginosa* and 53.0 µg/mL against *Staphylococcus aureus* at 4 nm	Human L929 fibroblasts	96% cell viability observed at 4 mM	Full-thickness excisional wound model in rats	Accelerated wound healing, earlier development, and maturation of granulation tissue	2020	[88]
Hydroxypropyl methylcellulose	40–70 nm	10, 20, 40, 80 µg/mL	-	Inhibition zone increased with increasing concentration. At 80 µg/mL: *E. coli*: 20 mm; *S. aureus*: 19 mm	-	-	Full-thickness excisional wounds in rats	The percentage wound healing for formulation was 9.34% more than that of standard (silver sulfadiazine cream) at day 14 with accelerated wound contraction and reduced epithelialization periods, however, the standard showed 1.78% higher healing on day 21.	2014	[89]
Zwitterionic poly (sulfobetaine methacrylate) monomer and protected dopamine methacrylamide monomers (DMA) hydrogel	18 ± 2 nm	2 mM	46 μg/L for a 5 × 5 cm^2^ sample during the first day and gradually increased thereafter	Inhibition Zone: 157, 148 and 129% for *E. coli*, *S. aureus*, and *P. auregenosa* compared to control. Less significant effect on Gram-positive than -negative bacteria. Suspension assays measuring the optical density (OD) of bacteria at 600 nm show significantly decreased OD values in AgNP group in comparison to other samples, demonstrating bactericidal effects	MC3T3-E1, Human HS68/F3T3 Fibroblasts	Due to the low concentrations of silver released, Mammalian cell viability was not greatly affected during 5 days of incubation	Full-thickness excisional wounds in rats	The percentage of the wound size reduction was 59% for the control, 80 for the hydrogel alone, and 98% for the AgNP-hydrogel treatment	2016	[90]
Pluronic F127	2.6 nm	200 µg/g	Initial burst during the first 10 h, followed by a sustained release over 24 h	AgNPs reduced *S. aureus* viability by up to 77% compared to 50% in SSD and 20% in the blank hydrogel Zone of Inhibition: AgNP hydrogel: 14 mm; SSD: 11 mm; blank hydrogen: 7 mm	-	-	*S. aureus* infected full-thickness excisional wound model in	AgNP hydrogel to the wound provides superior bactericidal activity and reduces inflammation leading to accelerated wound closure when compared to industry-standard silver sulfadiazine. It also accelerated wound closure and improved wound re-epithelialization. Further, decreased neutrophil infiltration, increased anti-inflammatory Ym-1 positive M2 macrophages, and reduced the number of caspase-1 positive apoptotic cells were also observed.	2021	[2]
Chitosan and Konjac Glucomannan	60 nm	200 µg/mL	Release in a gradual manner	Superior ability for AgNPs-loaded hydrogels to kill *S. aureus* and *E. coli.*	L929 cells	95% cell survival rate	*S. aureus* infected full-thickness wounds in rats	Promotes accelerated infected wound repair with no adverse reactions or symptoms	2020	[91]
Riclin	25.92 nm	1.0 mM	Sustained released with complete release observed at 48 h	Inhibitory concentration of *S. aureus* was 10 μg/mL and *E. coli* was 10 μg/mL	Mouse skin fibroblast (NIH3T3) cells and macrophage (Raw 264.7) cells	Weak cytotoxicity (>60% cell viability) toward NIH3T3 mouse fibroblasts at concentrations of 54 μg/mL	*S. aureus* infected full-thickness wounds in mice	Faster wound healing, more complete re-epithelialization, and denser collagen deposition characteristics.	2022	[92]
Chitosan	-	6, 12, 24, 48 mM (24 being the optimum dose)	After 12 h co-culture, concentrations of 3.01, 3.92, 6.62, and 8.26 mg/L were obtained with Ag^+^ doses of 6, 12, 24, and 48 mM, respectively	Significant bactericidal effects noted MPI: *S. aureus*: 35% and *S. epidermis:* 34%	NIH/3T3 cells and KERTr cells	Cell viability could be maintained >90% when the concentration of Ag^+^ in the HTM was <6 mg/L	Full-thickness wounds in diabetic rats	Higher wound closure efficiency and faster recovery of integrity and functionality of the newly formed tissues compared to other treatments	2021	[93]
Gelatin	300 nm	1 mg/mL	Quick release during the first 48 h, reaching 29.65% In the following stage, prolonged-release profiles over 504 h (21 days) were observed, with a cumulative percentage of 61.37%	A sustained antibacterial effect was observed against *E. coli* and *S. aureus* compared to no bacteriostatic ability in the pure hydrogel alone.	MC3T3-E1	Good cell compatibility observed	Scalded skin model to produce 2-degree burns in rats	Only 15% of the wound area left on day 10. Histology results showed the epidermal and dermal layers were better organized compared to the control.	2022	[94]
Lignocellulose	-	0.5 and 0.8% *w*/*v*	pH-dependent release	4.1% survival rate for *S. aureus* and 2.9% survival rate for *E. coli*	L929 fibroblast cell line	>95% of the cells are viable after 36 h incubation. No hemolytic activity observed.	*S. aureus* infected full-thickness wounds in mice	Significantly accelerate tissue regeneration and wound healing process through increasing collagen deposition and decreasing inflammation while retaining excellent biocompatibility	2021	[95]
Cellulose and gelatin	-	0.2 and 0.5 mg/mL	-	Decrease in *S. aureus* and *P. aeruginosa* activity Inhibition zone: ~2 mm at 0.5 mg/mL	Neonatal human dermal fibroblasts (NHDF)	CNF/G/Ag0.5 presented highest satisfactory infected cell viability (>100%)	Full-thickness wounds in mice	The CNF/G/Ag groups had much declined size of the wound than the control; wounds treated with CNF/G/Ag0.5 healed ∼90% after treated Bacterial infection of the wound was reflected by weight loss. Treatment with CNF/G/Ag0.5 displayed a clear advantage in survival rate (83.3%)	2018	[96]
Aloe vera-silk fibroin composite	40 nm	0.5 mg/mL	pH-dependent release: 40.89% release in neutral environment, and 55.12% in acidic environment	Antibacterial rings presented in the AgNP hydrogel had the largest diameter both for *E. coli* (13.92 ± 0.94 mm) and *S. aureus* (10.623 ± 0.61 mm), demonstrating superior antibacterial properties	L929, Mesenchymal Stem Cells	Promotion of cell proliferation and migration; good biocompatibility	Full-thickness excisional wounds in rats	Accelerating healing and inhibition of immune reactions observed with better performance in early inflammatory response stages. Good antibacterial properties, satisfactory biocompatibility and promotes cell proliferation, migration, and wound healing in the AgNP hydrogel compared to the controls.	2021	[97]
Cellulose	119.7 ± 5 nm (natural cashew gum—NCG); 123.8 ± 8.9 nm (phthalated cashew gum—PhCG)	NCG: 36 × 10^10^ particles/mL PhCG: 4.03 × 10^10^ particles/mL	-	Antibacterial activity was tested against *S. aureus* and *P. aeruginosa*. The hydrogel base alone did not present an antimicrobial effect. The effect of the hydrogels was more effective *against P. aeruginosa*, whereas the PhCG-AgNP was more potent than the NCG-AgNPs. For the gram-negative bacterium, the MIC values presented the same value of MBC for both hydrogels, which indicates a bactericidal effect. Hydrogels with AgNPs showed lower MICs when compared to the effect of AgNO_3_ solutions at the same concentrations tested for the two bacteria.	-	-	Full-thickness wounds in rats	Improved healing was observed compared to the control	2017	[98]
Cellulose	28 nm	-	2.0% *w*/*v*	Good resistance against Gram-positive and Gram-negative bacteria, with *E. coli* and *S. aureus* showing superior colony formation suppression. Bacterial death was recorded in 78.9 ± 2.61% of the cases in the control and 95.6 ± 1.93% in the presence of AgNPs	-	-	*S. aureus* infected full-thickness wounds in rats	Accelerated wound healing with superior antibacterial and wound healing properties noted. Significantly improved wound closure by day 16, and histological examination of the tissue in the wounded area showed rapid reepithelialisation, differentiated dermis, and epidermis, with minimal scar tissues.	2022	[99]
Silk fibroin	-	5% *w*/*v*	Rate of release of AgNP varies depending on metformin-loaded mesoporous silica microspheres (MET@MSNs): Ag NPs mass ratios	Colony counts reduced from 7.72 ± 0.10 (CFU/mL) to 6.90 ± 0.09 (CFU/mL) for *S. aureus* and from 7.15 ± 0.09 (CFU/mL) to 6.30 ± 0.43 for *E. coli*. Zones of inhibition for *S. aureus* and *E. coli* are comparable to antibiotic-sensitive tablets	RAW264.7, EA.hy926, and L929 cells	For RAW264.7 cells, >90% cell viability on day 7 with hydrogel application	Full-thickness excisional wounds in diabetic mice	Rapid wound healing was observed regeneration of squamous epithelium, collagen formation, and angiogenesis indicative of good wound repair compared to control	2022	[100]
Carbopol	21 nm	100 µg/g	-	Microbicidal activity on *S. aureus* and *E. coli* with MBC close to 100 µg/ mL, and MtE has a microbicidal response on *S. aureus* with MBC of 50 µg/mL. Besides, AgMt NPs-G produces a marked bacterial inhibition by contact in both strains	HUVEC cells	MtE and AgMt NPs tested concentrations for toxicity do not show an important effect on cell viability, except for AgMt NPs 100 µg/mL concentration, where cell viability falls by almost 10%	Second-degree burn injuries in rats	Higher wound healing ratio and faster wound evolution compared to control.	2021	[101]
Chitosan and polyethylene glycol	99.1 ± 2.3 nm	-	0.0055 µg/mL/h	AgNP-impregnated chitosan hydrogels have better antimicrobial potential compared to bare chitosan hydrogel and AgNPs. The zone of inhibition recorded for AgNP-loaded hydrogel against *E. coli*, *P. aeruginosa, B. subtilis* and *S. aureus* were 20.2 ± 1.0, 21.8 ± 1.5, 15.5 ± 0.8 and 21.5 ± 0.5 mm, respectively, which were higher compared to the controls	-	-	Full-thickness excisional wounds in diabetic-induced rats	The combination of AgNPs and chitosan hydrogel significantly enhances healing. Accelerated wound contraction as well as improved antimicrobial and antioxidant properties were observed compared to the controls.	2019	[102]
Carbopol	20 nm	0.18 mg/g	After 24 h, 10.56 µg/cm^2^ of AgNPs were released into the skin ex-vivo	-	Murine macrophage (J774A.1), human skin fibroblasts (TE 353.Sk) and human keratinocytes (HaCaT) cell lines	Good cellular uptake; no toxicity 48 h upon exposure	Full-thickness excisional and incisional wounds in rats	Accelerated and enhanced wound healing via modulation of cytokines and growth factors	2017	[103]
Chitosan and dextran	-	47 µg/g	The concentration of silver ion released was the highest at the 2 h time point and then declined thereafter	Almost all bacteria (*S. aureus* and *P. aeruginosa*) were dead after being treated with the hydrogel/NP after 60 min	NIH 3T3 cells	>80% viability	*S. aureus* and *P. aeruginosa* infected full-thickness excisional wounds in diabetic rats	Rapid wound contraction was observed after treatment with the hydrogel, suggesting superior healing activity to promote fibroblast migration, granulation tissue formation, and promotion of angiogenesis.	2019	[104]
Hyaluronic acid and gelatin	-	100, 200, 300 µg/mL	-	The bactericidal rates of both *E. coli* and *S. aureus* reached > 90% when the AgNPs concentration was 200 μg/mL. The area of the inhibition zone becomes larger with an increase in the AgNPs concentration.	L929 fibroblasts	Good cytocompatibility (cell viability >80% at 24 and 48hr)	Full-thickness excisional wounds in rats and abdominal wall model	The AgNP hydrogel accelerated the healing process by improving wet adhesion, reducing wound inflammation, promoting angiogenesis and formation of granulation tissues compared to the control.	2022	[105]
Chitosan, ulvan dialdehyde, human umbilical cord mesenchymal stem cell powder	-	5, 10, 20, 30, 40, 50 mg/mL	-	Zone of inhibition for pure hydrogel was 14 mm and 20 mm for *S. aureus* and *E. coli* respectively, compared to 23 mm and 31 mm for AgNP hydrogel	NIH-3T3 fibroblasts	Cell viability >80% with all concentrations	Full-thickness excisional wounds in Type II diabetic mice	Accelerated wound healing observed compared to control	2022	[106]
Polyvinyl alcohol and chitosan	-	5 mM	Optimal release at pH 5.7 compared to 7.4. Fastest release in first 3 days, tapering off to ~0.6 ug/mL by day 10.	The AgNP hydrogel demonstrated good bacteriostatic effect after 24 h incubation for both *E. coli* and *S. aureus* compared to the pure hydrogel which had almost no bacteriostatic effect.	L929	No changes in cell viability with L929 cells treated with AgNP hydrogel after day 1, decreased to ~90% by day 2, and ~80% by day 3.	*S. aureus* infected full-thickness excisional wounds in mice	Accelerated wound contraction and granulation thickness after 10 days compared to control. TNFalpha expression was lowest and VEGF expression was highest in hydrogels containing AgNP in irradiated mice treated with H_2_O_2_ in conjunction.	2022	[107]
Polyvinyl alcohol and alginate	30–40 nm	3.18 µg/mL	-	Hydrogels containing Ag-NPs effectively inhibited *E. coli* and *P. aeruginosa* from growing. A stronger response was seen with *P. aeruginosa* than *E. coli*.	RAW 264.7, human keratinocyte and human dermal fibroblast cell lines	Marginal cell toxicity due to low concentration of AgNP used	Full-thickness excisional wound model in rats	Accelerated wound closure with ameliorated inflammation, enhanced angiogenesis, increased collagen production, and promotion of re-epithelialization compared to control	2019	[108]
Polyacrylic acid sodium, polyvinyl butyral and chitosan	-	100, 200, 300 mg/L	The two-stage dressing released < 20% during first 30 min followed by faster release in greater quantities in subsequent period	-	-	As the nano-Ag concentration increased to over 300 mg/L, cell viability was reduced.	*S. aureus* infected full-thickness excisional wounds in diabetic mice	Accelerated wound healing and reduction of bacteria as well as promotion of re-epithelialization compared to control	2017	[109]
Polyvinyl alcohol and cellulose	-	0, 1, 2, 4 mM	-	The bactericidal rates of the AgNP hydrogel at a lower concentration against *E. coli* and *S. aureus* were 65.63 ± 2.63% and 51.17 ± 1.49%, respectively. At a higher concentration, the rates were 99.72 ± 0.14% and 99.38 ± 0.48% against *E. coli* and *S. aureus*, respectively	L929 cells	The cell survival rate slowly increased with prolonged culture time and ranged from 96% to 134%	Full-thickness excisional wound model in mice	The AgNP hydrogel promoted the growth and development of new blood vessels and significantly accelerated wound healing with their combined antibacterial and anti-inflammatory activities	2021	[110]
Hyaluronan-polyacrylamide	-	50, 100, 200 µg/mL	At the first stage, the release of Ag^+^ showed an exponential increase for the first ten days, followed by a ‘stationary’ phase that continued until the 30th day.	The highest antibacterial effect was exhibited at 200 µg/mL < 1% of the bacteria (*E. coli* and *S. aureus*) survived, compared with the control group. No inhibition zone is found in the 0 µg/mL group, whereas clear inhibition zones were observed for the 50, 100, and 200 µg/mL groups towards *E. coli* and *S. aureus*	3T3 cells	Comparable cell viability compared to control after being cultured for 24 h	*S. aureus* infected full-thickness excisional wound model in rats	The AgNP hydrogel significantly promoted wound healing by improving granulation tissue-formation, angiogenesis, and collagen deposition as well as alleviating inflammation.	2020	[111]
Hyaluronic acid	100 nm	0.5 µM	90% release within 48 h when the pH was reduced from 7.4 to 5.0	Robust antibacterial ratios for both *S. aureus* (95.69%) and *P. aeruginosa* (86.76%) compared to control	HUVECs and L929	>75% cell viability maintained over 3 days	*S. aureus* infected full-thickness excisional wound model in diabetic rats	Accelerated wound closure as well as increased anti-inflammatory, pro-angiogenic, and antibacterial activities were seen in the hydrogel compared to the control.	2022	[112]
Polyvinyl alcohol and gelatin	-	0.1, 0.2, 0.3, 0.4 mM	-	Against *E. coli* and *S. aureus*, a larger inhibition zone compared to the pure hydrogel.	HaCat, LO2 and 293T cells	No inhibitory effects seen, and at low concentrations, a tendency for cell proliferation was noted (5, 10, 15 µg/mL). At higher concentrations, low inhibition was seen, <20% (20, 30, 50, 100 µg/mL)	*S. aureus* infected full-thickness excisional wounds in mice	Accelerated wound healing, anti-bacterial properties, reduced inflammation, and increased collagen content compared to control	2021	[113]
Propyl methacrylate	-	140, 280, 420 µg/mL	The release amount of Ag^+^ in Ag_2_S QDs group was much higher than that in NP hydrogel treatment group. Compared with the NP hydrogel-NIR (−) group, the NP hydrogel-NIR (+) group showed a larger release amount of Ag^+^ (39.9–84.9 ppb versus 36.6–73.9 ppb), indicating that NIR could accelerate the release of Ag^+^. NIR = near-infrared radiation	The inhibition zone diameter of the NP hydrogel group (NP hydrogel-NIR (+) and NP hydrogel-NIR(−)) was significantly larger than that of the control group, with a positive correlation with the NPs concentration against *E. coli* and MRSA. Additionally, the inhibition zone diameter was obviously larger in the NP hydrogel-NIR (+) group than the NP hydrogel-NIR (−) group, demonstrating that the antibacterial ability of the NP hydrogel was enhanced under the assistance of NIR laser irradiation.	NIH 3T3 MEFs, Vero cells	At 420 µg/mL NP concentration, cell survival was still as high as 93.8 ± 3.7% for Vero cells and 96.8 ± 6.2% for NIH 3T3 cells after 48 h of incubation, demonstrating good cytocompatibility	MRSA infected full-thickness excisional wounds in mice	9 days of treatment with NP hydrogel could heal the full-thickness skin defects infected with MRSA with enhanced bacterial clearance, significant collagen deposition, upregulation of VEGF expression, and angiogenesis at the infected sites.	2022	[114]
Copolymer (PEP)	-	0.75% *w*/*v*	-	Good antimicrobial activity against MRSA and *E. coli* with inhibition zone of 1.3 cm and 1.4 cm, respectively	HUVECs, NIH 3T3 MEFs	for HUVEC, cell viability >98% for 200 µg/mL; for NIH-3T3 cells, cell proliferation was not inhibited over a 3-day span for varying concentrations (50–200 µg/mL)	MRSA infected full-thickness excisional wounds in rats	Rapid wound healing—99.85% of the aggregate wound area was healed over the span of 12 days whereas only 54% wound closure was observed for the untreated group.	2019	[115]
Chitosan	60–150 nm	0.5–6.0 mM	-	More obvious zone of inhibition for *S. aureus* compared to *E. coli*. The pure hydrogel alone demonstrated no antibacterial activity	L929 cells	Good cell viability (>90%) for all hydrogels tested	*S. aureus* infected full-thickness excisional wounds in rats	Accelerated the healing process through anti-infection, anti-inflammation, stimulating collagen deposition, and promoting the formation of epithelia and blood vessels compared to control	2022	[116]
Methacrylate gelatin	120 ± 3.392 nm	20 µg/mL	Day 7, the Ag^+^ release ratio of the hydrogel was 64.2% ± 4.3% and 70.7% ± 7.8% in solution containing either lysozyme or not, respectively	The numbers of bacterial colony-forming units (CFU) were reduced to 75.3% ± 0.8% for *E. coli,* 88.8% ± 1.3% for *S. aureus*, and 82.1% ± 1.4% for *P. aeruginosa*	NIH 3T3	Excellent biocompatibility observed	*E. coli* and *S. aureus* full-thickness skin burn model in rats	Promotes wound healing by facilitating the regeneration of the epithelial wounds, protecting the wound-rebuilding microvessel network, reducing the inflammation-induced infiltration, enhancing the collagen deposition, and inducing the macrophages to the anti-inflammatory phenotype with noncanonical Wnt signal pathway activated	2022	[117]
Gelatin and polyvinyl alcohol	7.4 ± 1.2 nm	-	Sustained release of silver from the hydrogel was detected, and an accumulative 8.99 0.58% of total silver was released after 24 h and 14.98 0.71% was released after 72 h.	>99% of inhibition in all three bacterial strains (99.91 ± 0.52% for *E. coli*, 99.89 ± 0.35% for *S. aureus*, and 99.57 ± 0.73% for MRSA)	L929 cells	Similar biocompatibility compared to pure/blank hydrogel	Full-thickness wounds in rats	Improved antibacterial efficacy, accelerated wound healing and rapid re-epithelialization compared to control	2022	[7]

**Table 2:** Summary of Characteristics and Findings of Included Trials for Gold Nanoparticles.

Hydrogel Species	Particle Size (Diameter)	Dosage of NP	Rate of Release	Antimicrobial Capability	Cell Type	Cytotoxic Effect	In Vivo Model	In Vivo Findings	Year	Source
*Cydonia oblonga* seed extract	20–30 nm	10 mmol	-	MIC: *B. simplex*: 16 mg/mL; *B. subtilis:* 32 mg/mL; *P. aeruginosa:* 32 mg/mL; *E. coli*: 16 mg/mL; *S. aureus:* 40 mg/mL; *A. niger:* 50 mg/mL; *P. notatum*: 50 mg/mL Inhibition zone: *B. Simplex:* 15 mm; *B*. *Subtilis:* 17 mm; *P. aeruginosa*: 16 mm; *E. Coli:* 18 mm; *S. Aureus*: 12 mm	-	-	Full-thickness wounds in mice	99% wound closure in 5 days; increased expression of NANOG and CD-4 in nanoparticle treatment group	2022	[133]
Alginate	25 nm (NP), 50, 70, 120 nm spike length (nanostars (NS))	1.5 µg/mL	NP: 157 ng release over 12 h; NS: 8.63 ng release over 12 h	The bacterial killing of >95% is observed for *P. aeruginosa* and *E. coli*, while up to 60% for Gram-positive *S. aureus*. >80% of colonies of *P. aeruginosa* and *E. coli* were also reduced. 35.4% reduction of colonies were obtained for *S. aureus*.	NIH-3T3	85% viability	*S. aureus* infected full-thickness wounds in rats	Accelerated wound healing with enhanced wound closure and angiogenesis	2022	[134]
Poloxamer	29.2 ± 2.1 nm	-	Slow and prolonged release over 48 h, ~2%/min	High percentage reduction of bacterial viable count against *S. aureus* and *P. aeruginosa*	-	-	Full-thickness excisional wound model in rats	Almost completely healed wound after 14 days of daily treatment compared to control, owing to their enhanced skin re-epithelization effect and collagen formation, in addition to their impact on the gene expression of inflammatory and anti-inflammatory mediators. Furthermore, low percentages of deposition into the main body organs after 21 days of daily wound treatment was seen.	2019	[10]
Polyethyleneimine (PEI), polyethylene glycol (PEG), hexachlorocyclic triphosphonitrile (HCCP)	22 nm	0.3, 3, 5 nM	Release ratio of approximately 70% after 16 h incubation with the tendency to slow down thereafter	Improved performance against *S. aureus* and MRSA, especially if the gel is exposed to laser irradiation	L929 cells	Negligible cytotoxicity, good biocompatibility, and excellent hemocompatibility	Full-thickness excisional wound model in mice	Accelerated wound healing with no toxicity or significant adverse effects compared to the control.	2022	[135]
Alginate	-	0.05, 0.1, 0.2 mg/mL	In PBS (pH 7.2–7.4, 0.1 M), approximately 74%, 73%, and 76% of Ga^3+^ was released from Ga^3+^-crosslinked hydrogel	Strong bactericidal activity against *S. aureus* and *P. aeruginosa* observed with higher reduction in *P. aeruginosa* compared to *S. aureus*. Faster release of silver ions demonstrated stronger antibacterial effect.	Keratinocytes (HaCaT)	After 1, 3 and 7 days of incubation, the materials did not show any toxicity even after 7 days of contact and up to 96% of keratinocytes were viable	Full-thickness wounds in diabetic and non-diabetic mice	Rapid contraction of wound edges was seen in comparison to the controls as well as minor scab formation and lack of inflammation in the integument. Fifteen days of treatment with the nano-enabled hydrogels completely recovered the wounds of non-diabetic and diabetic mice. The bactericidal effect was also evidenced by absence of bacterial contamination in the wounds.	2021	[136]
Chitin	215.31 nm	2.5, 5, 10% *w*/*v*	-	Hydrogels with Au contents of 5% and 10% were most effective at inhibiting *E. coli* growth, whereas a content of >2.5% was sufficient to completely inhibit the growth of *S. aureus* colonies	L929 cells	The survival rate of cells in all concentrations of Au was >80% at 2 h, and even over >90% in most groups. At 48 h, except for the 10% group, the cell viability of other groups was >80%.	*S. aureus* infected full-thickness wound model in mice	Good antibacterial, hemostatic, and anti-inflammatory properties.	2023	[137]

**Table 3:** Summary of Characteristics and Findings of Included Trials for Copper Nanoparticles.

Hydrogel Species	Particle Size (Diameter)	Dosage of NP	Rate of Release	Antimicrobial Capability	Cell Type	Cytotoxic Effect	In Vivo Model	In Vivo Findings	Year	Source
Gelatin	-	0, 0.5, 1, 1.5 mg/mL	Rapid release over 5 days with stability between 5–7 days	After 9 h of incubation, the survival ratios of *S. aureus* and *E. coli* were 7.5% and 0.0%, respectively, compared to no antimicrobial activity in the control. The antibacterial properties of the hydrogels depended on the Cu^2+^ concentration, and a higher Cu^2+^ concentration led to a lower bacterial survival ratio.	Mouse L929 fibroblasts	Good biocompatibility	Full-thickness excisional wound model in diabetic mice	1 mg/mL hydrogel demonstrated regular epithelialization and collagen deposition compared to control as well as promotion of angiogenesis and reduction in proinflammatory factors	2022	[156]
Polyacrylamide	-	0 and 6% mole fractions	-	The bacterial viability of *E. coli* decreased from 26.8% to 5.1% with the increase from 2 mol% to 10 mol%	EC and SMC cells	Cell viability > 85%	*E. coli* infected full-thickness excisional wound model in mice	The hydrogel killed the bacteria and promoted the production and deposition of collagen, thus effectively reducing the inflammatory response as well as accelerating the healing of wounds in comparison to the control	2022	[157]
Chitosan and polyvinyl alcohol	50–100 nm	1 mM	Rapid release initially then constant release for up to 12 h	Showed inhibition against all tested microorganisms. The strongest activity was against *Bacillus cereus* (29.6 ± 0.42 mm), followed by *S. aureus* (15.6 ± 1.1), *E. coli* (13.3 ± 0.8) and then by *P. aeruginosa* (10 ± 1 mm).	-	-	Full-thickness excisional wound model in rats	Accelerated wound healing observed compared to control	1997	[158]
3-(tri-methoxysilyl) propyl methacrylate and mesoporous silica	25 nm	0.5, 1, 1.5 mg/mL	Slow release observed in the first 2 days, relatively rapid release in the subsequent 5 days, and stabilization afterwards.	Antibacterial efficacy of 99.80% and 99.94% against *S. aureus* and *E. coli* within 10 min after application, respectively under NIR light irradiation	NIH-3T3 cells (mouse embryonic fibroblast cell line)	Hydrogels with smaller concentrations of NPs do not exhibit appreciable negative effects on cell viability, demonstrating the good biocompatibility	Full-thickness excisional wound model in rats	The NPs-Hydrogel is demonstrated to have the ability to kill bacteria while promoting the healing of wounds.	2018	[159]
Alginate	50 nm	31.25 mM	Plateau of release at 24 h	The numbers of viable *S. aureus* and *E. coli* colonies were significantly decreased in the presence of CuNPs compared to the control, demonstrating superior antibacterial properties compared to conventional hydrogels alone.	Human umbilical venous endothelial cells (HUVECs)	>85% cell viability (low toxicity)	Full-thickness excisional wound model in diabetic mice	Increased levels of hypoxia-inducible factor 1 alpha and vascular endothelial growth factor; profound diabetic wound area reduction in 10 days (treatment group) vs. 14 days (control group)	2022	[160]
Gelatin	110 nm	1, 2, 3, 4, 8 mM	-	As compared to bare hydrogels, the viabilities of *E. coli* and *S. aureus* reduced from 92.4% to 8.8% and 93.7% to 9.2%, respectively, when the concentrations of Cu NPs added to hydrogel samples increased from 1 mM to 8 mM for an incubation time of 6 h.	NIH-3T3 cells	No obvious effect on the proliferation of NIH-3T3 cells. No change measured in inflammatory gene expression.	*S. aureus* infected full-thickness excisional wound model in rats	Prominent wound closure of up to 95.1%, while the control group wound closure was around 79.3%.	2019	[161]
Polydopamine	-	1.25, 2.5, 5 ppm	Cu ion concentration (ppm) was released over 12 h in hydrogel. Concentration was higher after 12 h when the hydrogel was irradiated (3.0 ppm) compared to not (~2.0 ppm)	Highest rates of inhibition against MRSA and *E. coli* were observed when the CuNP hydrogel was combined with laser irradiation (>90% for both strains)	HDFs and HUVECs	No cytotoxicity, cell proliferation was observed after 5 days	*E. coli* infected full-thickness excisional wound model in rats	Improved wound healing and collagen deposition observed. The CuNP hydrogel promoted cell proliferation and angiogenesis. Wound closure was greatest when hydrogel was combined with irradiation after 14 days	2020	[162]
Chitosan	3–5 nm	100, 200, 400 µg/mL	-	Good antibacterial activity against *S. aureus* and *E. coli* (kills 96.5% of *S. aureus* and 98.40% *E. coli*)	NIH 3T3	Cu hydrogel with 808 nm laser irradiation has cytotoxic effects (54.6% cell viability). After 1 week of cell culturing, cell viability is 88.4% for Cu hydrogel with no laser irradiation. Positive charge on chitosan is cytotoxic.	Full-thickness skin defect model in mice	Enhanced wound healing; 15% smaller wound size was observed for Cu hydrogel (+808 nm) group at the 7-day mark compared to control; biocompatible since no major cytotoxic effects on major organs based on histological analysis	2021	[163]
Sericin, chitosan, and polyvinyl alcohol	7 nm	-	-	Against drug-sensitive *S. aureus*, *E. coli*, and drug-resistant MRSA, the hydrogel inactivated the bacteria in their respective regions, resulting in no significant change in the size of the inhibition zone for 7 days	L929	The hemolysis rate of the composite was 3.9% and the cell viability rate was 131.2%; good biocompatibility	Full-thickness excisional wounds in mice	Accelerate wound healing and re-epithelialization were observed compared to the control	2021	[164]

**Table 4:** Summary of Characteristics and Findings of Included Trials for Copper Oxide Nanoparticles.

Hydrogel Species	Particle Size (Diameter)	Dosage of NP	Rate of Release	Antimicrobial Capability	Cell Type	Cytotoxic Effect	In Vivo Model	In Vivo Findings	Year	Source
Starch	30–50 nm	2% and 4% *w*/*v*	-	20–32 mm inhibition zone of Gram-positive and negative organisms	Human fibroblasts	Higher toxicity with higher concentration due to the generation of ROS	Full-thickness wounds in rats	Accelerated wound healing	2021	[165]

**Table 5:** Summary of Characteristics and Findings of Included Trials for Zinc Nanoparticles.

Hydrogel Species	Particle Size (Diameter)	Dosage of NP	Rate of Release	Antimicrobial Capability	Cell Type	Cytotoxic Effect	In Vivo Model	In Vivo Findings	Year	Source
Chitosan	-	0.5% *w*/*v*	Constant drug release properties	The disruption rate for *S. aureus* biofilms was 59.10% at 24 h and 25.64% at 48 h and was significantly lower than those for other bacteria. By comparison, the disruption was excellent for *P. aeruginosa* (81.97% at 24 h, 79.43% at 48 h) and mixed strains (93.64% at 24 h, 74.91% at 48 h). No difference was observed in the size of inhibition zone when ZnG was added.	NIH/3T3s	No toxicity was observed against NIH/3T3s when co-incubation with 40% and 70% of ZnG/rhEGF@Chit/Polo in the growth medium.	Scald wounds in rats	Supplements wound healing to promote the vascular remodeling and collagen deposition, facilitates fibrogenesis, and reduces the level of interleukin 6 for wound basement repair, and thus is a good wound therapy.	2021	[170]
Alginate	-	10, 20, 40 mM		Antibacterial rate was 100% in contrast to the control which did not show any inhibitory effect on *E. coli* growth.	Human dermal fibroblasts and human umbilical vein endothelial cells	Cell proliferation and good cytocompatibility was observed.	Full-thickness excisional wound model in mice	The wound-healing process was enhanced, and the formation of epithelium and blood vessels were observed in the NP group compared to the control.	2017	[171]
Alginate	-	0.1 M	11 µg/mL over 60 h (~60–80% cumulative release)	Good bacterial inhibition against both *E. coli* and *S. aureus* that was even stronger in Alg@Zn hydrogels with increased concentrations of CBD versus without	NIH 3T3 cells and HUVECs	Good cell viability (>80%) for both cell lines, measured on days 1, 3, and 5.	Full-thickness excisional wound model in rats	The ZnNP hydrogel facilitated wound healing, controlled inflammatory infiltration, promoted collagen deposition, granulation tissue, and angiogenesis	2022	[172]
Poly-sulfobetaine methacrylate	-	-	-	Survival rate of *S. aureus* and *E. coli* upon the application of the ZnNP hydrogel is 0	L929 cells	Good biocompatibility	*E. coli* and *S. aureus* infected full-thickness excisional wounds in rats	Accelerated and ameliorated infected healing (quantitative analysis shows better wound healing for *E. coli* infected wounds compared to *S. aureus* infected wounds).	2022	[173]
Gelatin methacryloyl	45 nm	-	ZnS NPs were constantly released for at least 15 days (~60 ug/mL at day 14)	-	L929 cells	no differences in cell viability between Zn hydrogel and control (measured on days 1, 3, 5)	Full-thickness excisional wounds in rats	Effective and accelerated wound healing (near 100% by day 15 compared to ~70% for the control patch)	2022	[174]
Glucose oxidase with deferoxide mesylate to promote synergistic antibacterial effect	-	-	The concentration of Zn in the DG@Gel group reached 17.05 mg/mL at 24 h significantly higher than the concentration of the Gel group (10.94 mg/mL).	In contrast, DG@Gel exhibited distinct inhibition zones with diameters of 24.40 ± 2.43 mm and 12.15 ± 0.64 mm for *S. aureus* and *E. coli*, respectively	HUVECs and NIH/3T3 cells	No cytotoxicity	Full-thickness excisional wounds in diabetic mice	Anti-bacterial, angiogenic activity for diabetic wound repair, re-reepithelialisation and collagen deposition compared to control	2022	[175]
Histidine and sodium alginate	-	10 and 50 mM	-	Good antibacterial effect toward *E. coli* and *S. aureus* 98.2 ± 1.1%	NIH3T3 cells	Insignificant cytotoxicity after being co-incubated with NIH3T3 cells for 1, 3, and 5 days	*S. aureus* infected full-thickness excisional wounds in diabetic mice	Accelerated wound healing with full repair seen in 13 days compared to 27 days in control, as well. Better healing performance also seen compared to control with anti-inflammatory effects, increased collagen deposition and promotion of granulation tissue deposition	2022	[176]
Chitosan	-	0.5, 1, 2% *w*/*v*	-	>90% antibacterial effect against *S. aureus* and *E. coli*, respectively	L929 and HUVEC	Good biocompatibility, promoting cell proliferation and migration	*S. aureus* infected full-thickness excisional wounds in mice	The ZnNP hydrogel promoted collagen deposition, reduced inflammatory expression, demonstrated hemostatic properties, and promoted wound healing.	2022	[177]

**Table 6:** Summary of Characteristics and Findings of Included Trials for Zinc Oxide Nanoparticles.

Hydrogel Species	Particle Size (Diameter)	Dosage of NP	Rate of Release	Antimicrobial Capability	Cell Type	Cytotoxic Effect	In Vivo Model	In Vivo Findings	Year	Source
Chitosan	27 ± 9 nm	0.1 mg/mL	-	MIC: *S. aureus*: 50 μg/mL for both HP-nZnO and non-functionalized nZnO. *E. coli:* HP-nZnO and nZnO was 100 and 200 μg/mL, respectively Inhibition Zone: *S. aureus:* ~34–39 nm; *E. coli*: ~33–38 nm	L929 and HDF cells	All hydrogel samples had a cell viability > 70%; with time from 24 to 48 h, cell viability increased. The presence of ZnO decreased cell viability slightly	Full-thickness excisional wounds in rats	Accelerated wound closure, re-epithelialization and decreased collagen deposition compared to other groups	2021	[178]
Methylated kapp-carrageenan	171–279 nm	0.5, 1, 2% *w*/*v*	-	The incorporation of ZnO/PD nanoparticles in KaMA hydrogel matrix increased antibacterial activity dramatically. The inhibition zone was four times wider than that of the control; however, the inhibition zone against *E. coli* bacteria was approximately 2-fold greater than that of *S. aureus*.	L929 fibroblast cells	>95% cell viability after 3 days of incubation as well as biocompatibility and blood clotting ability	Full-thickness excisional wounds in rats	Accelerated wound healing without any infection and superior granulation tissue thickness than control.	2020	[179]
Chitosan and sodium alginate (SA)	24.9 nm	-	ZnO NP released was tracked over 150 h, in hydrogel with two different concentrations of SA (47% and 33%). both hydrogels exhibited good release of ZnO (~60% and 50% by 150 h)	Inhibition zones of SA-COS-ZnO hydrogel were higher for all strains compared to control gel against *E. coli, S. aureus, C. albicans, B. subtilis.* B. subtilis inhibition zone was largest (1.5 cm to 3.1 cm)	3T3 cells 293T cells	The cell viabilities of all hydrogels were above 80%, and the viability increased with the increase of hydrogel concentration, indicating that the hydrogel had good biocompatibility.	Second-degree scald burn model in rats	Hydrogel rate of wound healing was highest (89.9%) on day 19 compared to 73.1 and 76.9% for the control groups. Hydrogel treatment resulted in higher collagen deposition, formation of hair follicles, blood vessels, and sebaceous glands	2021	[180]
Alginate	200 nm	0.01, 0.02, 0.04% *w*/*v*	-	The trend of the antibacterial rate against *E. coli* and *S. aureus* increases gradually with improving the content of ZnO in the hydrogel films, and a maximum antibacterial rate of 68.4% can be attained when the content of ZnO is 0.04%	L929 cells	No significant cytotoxicity	Full-thickness excisional wounds in rats	Accelerated wound healing compared to control.	2019	[181]
Epigallocatechin-3-gallate	-	-	-	Effectively cleared 95.6% of MRSA and 97% of *E. coli* with good bactericidal activity	HFF-1, HaCaT, and HUVECs	Good cytocompatibility with no significant toxicity	*E. coli* infected full-thickness skin wound model in diabetic rats	Accelerated wound healing by reducing inflammatory response and promoting proliferation of vascular endothelial cells and skin epidermal cells. After 15 days, the rate of skin lesion closure was 96.3%, compared to 65.4% in the control	2022	[182]

**Table 7:** Summary of Characteristics and Findings of Included Trials for Manganese Nanoparticles.

Hydrogel Species	Particle Size (Diameter)	Dosage of NP	Rate of Release	Antimicrobial Capability	Cell Type	Cytotoxic Effect	In Vivo Model	In Vivo Findings	Year	Source
Polydopamine and acrylamide ± glucose oxidase (GOx)	10 nm	-	-	The bactericidal efficiencies of MnO_2_ hydrogel + NIR and GOx/MnO_2_ hydrogel + NIR treatment groups for *E. coli* and *S. aureus* were 97.70% and 99.99%, 97.87% and 99.99%, respectively.	L929 cells	>95% viability after 24, 48, and 72 h of incubation	Full-thickness excisional wounds in diabetic mice	GOx/MnO_2_ hydrogel + NIR group showed accelerated healing and vascular regeneration compared to control	2022	[185]
Dextran, dopamine, carbodiimide	10 nm	-	-	Bactericidal efficiency 97.22% and 99.99% against *E. coli*—and *S. aureus*, respectively	L929, erythrocytes	Hemolysis ratio <2%; good cell viability after 72 h incubation	Full-thickness excisional wounds in diabetic mice	Complete wound healing observed over a 2-week period. Effectively supply O_2_ by converting the endogenous H_2_O_2_ into O_2_ in the wound microenvironment, as well as reduce inflammation, accelerate collagen deposition, and promote angiogenesis.	2022	[186]

**Table 8:** Summary of Characteristics and Findings of Included Trials for Iron Nanoparticles.

Hydrogel Species	Particle Size (Diameter)	Dosage of NP	Rate of Release	Antimicrobial Capability	Cell Type	Cytotoxic Effect	In Vivo Model	In Vivo Findings	Year	Source
Gelatin	-	12–16 mM	-	90% kill rate against *S. aureus* and *E. coli*	Mouse fibroblast cells (L929)	The relative cell viabilities of all tested samples were above 95%	*S. aureus* infected full-thickness excisional wound model in diabetic rats	Reduced infection and promotion of wound healing observed compared to control	2020	[190]
Polyacrylamide	-	5 mM	-	Antibacterial effect on *S. aureus* reached nearly 100% under the combined action of H_2_O_2_ and 808 nm near-infrared (NIR) laser	HEK293 cells & 3T3 fibroblast cells	HEK293 cells—cell viability >90% when FePDA concentration < 300 micrograms/mL (low toxicity); 3T3 fibroblast cells demonstrated cell proliferation	*S. aureus* infected full-thickness excisional wound in mice	Lowest relative wound area and optimal wound healing within 5 days of treatment was found in the NP group, thereby indicating the intensive skin wound disinfection	2022	[191]
Chitin and tannic acid	-	-	Released in a sustained manner	The 100% antimicrobial activity was due to the abundance of Fe^3+^ ions: 92% antibacterial activity against *E. coli* and 97% against *S. aureus* after 24 h	NIH 3T3 fibroblasts; erythrocytes	TA can be cytotoxic if present in excessive amounts; increasing ratio of Fe^3+^ ions improve cell viability	(1) Injection of *S. aureus* subcutaneously followed by injection of hydrogel and NIR Full-thickness excisional wounds in mice	Effective and rapid bactericidal effects were seen with 10 min of near-infrared laser irradiation. A combination of low-level laser therapy with hydrogel is conducive to the acceleration of wound closure and promotion of skin tissue repair.	2021	[192]
Chitosan	-	0, 2.25, 4.5, 9 mM	-	Against *E. coli* and *S. aureus*, >95% of both were killed by all hydrogel groups. All hydrogels with or without NIR irradiation showed an obvious zone of bacterial inhibition with diameters against *E. coli* and *S. aureus* of 1.2 cm (NIR+)/1.3 cm (NIR-) and 1.3 cm/1.3 cm in Fe0 group, 1.3 cm/1.3 cm and 1.3 cm/1.3 cm in Fe2.25 group, 1.5 cm/1.5 cm and 1.5 cm/1.5 cm in Fe4.5 group, 1.5 cm/1.5 cm and 1.5 cm/1.5 cm in Fe9 group, respectively.	L929	>80% viability over 2 days	*S. aureus* infected full-thickness excisional wounds in mice	More effective adhesive effects, antibacterial properties against *S. aureus*, angiogenesis, reduced inflammation, and decreased secretion of various proinflammatory cytokines was observed in the hydrogels compared to the control. Increased percent of M2 phenotype macrophages, and collagen deposition around the wound was also noted, thus accelerating wound healing and regeneration.	2022	[193]
Glycyrrshizic acid	-	1.0 mg/mL	Slow and sustained release observed	Fe^2+^/GA hydrogels exhibited high antibacterial capabilities against both *S. aureus* and *E. coli* in a dose-dependent manner compared to GA hydrogel alone	3T3 fibroblasts	No obvious cytotoxic effects observed; cell proliferation observed at 24 h incubation	*S. aureus* infected full-thickness excisional wounds in mice	Accelerated wound healing and antibacterial activity observed as well as anti-inflammatory properties compared to control.	2023	[187]

**Table 9:** Summary of Characteristics and Findings of Included Trials for Gallium Nanoparticles.

Hydrogel Species	Particle Size (Diameter)	Dosage of NP	Rate of Release	Antimicrobial Capability	Cell Type	Cytotoxic Effect	In Vivo Model	In Vivo Findings	Year	Source
Alginate	-	-	Constant for the first 3 days and then gradually declined with a decline in Ga concentration	87.79% *E. coli* and cells 88.76% *S. aureus* killed at 24 h Zone of Inhibition: 19 mm for *S. aureus* and 23 mm for *E. coli*	NIH3T3 cells	Low risk of hemolysis; good cell viability	*S. aureus* infected full-thickness skin wound model in mice	Accelerated infected wound healing with good biocompatibility, angiogenesis, enhanced fibroblast migration, and good collagen deposition observed compared to control	2022	[194]

**Table 10:** Summary of Characteristics and Findings of Included Trials for Combinations of Nanoparticles.

Nanoparticles	Hydrogel Species	Particle Size (Diameter)	Dosage of NP	Rate of Release	Antimicrobial Capability	Cell Type	Cytotoxic Effect	Animal Model	In Vivo Findings	Year	Source
Ag^+^ and Cu^+2^	Chitosan	-	-	75% Ag^+^ and 40%Cu^2+^ released over the first 2 days	Good antibacterial activity against *S. aureus* (4 mg/mL hydrogel concentration) and *E. coli* (2 mg/mL hydrogel concentration) Inhibition Zone: inhibition zones were present for the chitosan-Ag and chitosan-Ag-Cu groups at the 24 h mark	L929, HUVEC, RBCs	Hemocompatible (<5% hemolysis); Cell viability is good for concentrations < 4 mg/mL	*S. aureus* infected full-thickness excisional wound model in type 1 diabetic and normal rats	Accelerated healing; significant reduction in wound size at the 14-day mark	2022	[198]
ZnO and AgNP	Cellulose	20–40 nm AgNP	0.75, 1.25, 2.5 mM of AgNP	Sustained release over a period of 21 with initial short burst over 3 days	This hydrogel system kills 95.95% of *E. coli* and 98.49% of *S. aureus* within 20 min upon exposure to simulated visible light, and rapid sterilization plays a crucial role in wound healing. No inhibition zone is observed without NPs, whereas clear inhibition zones are formed around the nanocomposite hydrogels	Mouse calvarial cells (MC3T3-E1)	Ag NPs exhibit cytotoxicity at only small concentrations. However, lower cytotoxicity is seen with the AgNPs in the presence of ZnO	*S. aureus* infected partial thickness wounds in rats	In vivo results show that release of Ag^+^ and Zn^2+^ stimulates the immune function to produce a large number of white blood cells and neutrophils (2–4 times more than the control), thereby producing synergistic antibacterial effects and accelerated wound healing.	2017	[199]
Au and Pt	Hyaluronic acid and chitosan	3.6 ± 0.2 nm	50 μg/mL AuPt	In the first 6 h, the release rate of Au-Pt alloy NPs was 49%, and reached 84% within 36 h	The majority of *E. coli* (>95%) and *S. aureus* (>90%) were inactivated by the Au-Pt hydrogel	L929 cells	No statistically significant difference was observed between the control and the hydrogel-treated cells, therefore good biocompatibility	Full-thickness cutaneous wounds in diabetic rats	Accelerated and ameliorated wound healing via inhibition of inflammation, stimulation of angiogenesis and promotion of re-epithelialization and collagen deposition compared to control.	2022	[201]

## Data Availability

Not applicable.
